# Physical activity, sedentary time and gain in overall and central body fat: 7-year follow-up of the ProActive trial cohort

**DOI:** 10.1038/ijo.2014.66

**Published:** 2014-05-20

**Authors:** R Golubic, K Wijndaele, S J Sharp, R K Simmons, S J Griffin, N J Wareham, U Ekelund, S Brage

**Affiliations:** 1 Medical Research Council Epidemiology Unit, University of Cambridge School of Clinical Medicine, Institute of Metabolic Science, Addenbrooke's Hospital, Cambridge, UK; 2 Department of Sport Medicine, Norwegian School of Sport Sciences, Oslo, Norway

**Keywords:** moderate-to-vigorous physical activity, sedentary time, body fat, type 2 diabetes, family history

## Abstract

**Objective:**

The objective of this study is to examine the independent associations of time spent in moderate-to-vigorous physical activity (MVPA) and sedentary (SED-time), with total and abdominal body fat (BF), and the bidirectionality of these associations in adults at high risk of type 2 diabetes.

**Design and subjects:**

We measured MVPA (min per day) and SED-time (h per day) by accelerometry, and indices of total (body weight, fat mass (FM), BF% and FM index) and abdominal BF (waist circumference (WC)) using standard procedures in 231 adults (41.3±6.4 years) with parental history of type 2 diabetes (ProActive UK) at baseline, 1-year and 7-year follow-up. Mixed effects models were used to quantify the independent associations (expressed as standardised β-coefficients (95% confidence interval (CI))) of MVPA and SED-time with fat indices, using data from all three time points. All models were adjusted for age, sex, intervention arm, monitor wear time, follow-up time, smoking status, socioeconomic status and MVPA/SED-time.

**Results:**

MVPA was inversely and independently associated with all indices of total BF (for example, 1 s.d. higher MVPA was associated with a reduction in FM, *β*=−0.09 (95% CI: −0.14, −0.04) s.d.) and abdominal BF (for example, WC: *β*=−0.07 (−0.12, −0.02)). Similarly, higher fat indices were independently associated with a reduction in MVPA (for example, WC: *β*=−0.25 (−0.36, −0.15); FM: *β*=−0.27 (−0.36, −0.18)). SED-time was positively and independently associated with most fat indices (for example, WC: *β*=0.03 (−0.04, 0.09); FM: *β*=0.10 (0.03, 0.17)). Higher values of all fat indices independently predicted longer SED-time (for example, WC: *β*=0.10 (0.02, 0.18), FM: *β*=0.15 (0.07, 0.22)).

**Conclusions:**

The associations of MVPA and SED-time with total and abdominal BF are bidirectional and independent among individuals at high risk for type 2 diabetes. The association between BF and MVPA is stronger than the reciprocal association, highlighting the importance of considering BF as a determinant of decreasing activity and a potential consequence. Promoting more MVPA and less SED-time may reduce total and abdominal BF.

## Introduction

Type 2 diabetes mellitus (T2DM) and its complications constitute a major public health burden and account for considerable morbidity and mortality worldwide.^1^ Individuals with a family history of T2DM are 2–6 times more likely to develop the disease compared with those without.^2^ T2DM risk is additionally elevated by physical inactivity^3^ and weight gain.^4^ There is accumulating evidence that prolonged sedentary behaviour is an independent risk factor for weight gain, obesity^5,6^ and other chronic diseases such as cardiovascular disease^7^ and T2DM.^5,8,9^

Several prospective studies using self-reported physical activity (PA) have shown inverse associations between total PA and weight gain in the general population, but findings were inconsistent and effect sizes were small.^10^ Less attention has been given to individuals at increased risk for developing T2DM. Furthermore, abdominal fat has been shown to be a stronger predictor of several disease outcomes as compared with total body fat (BF), including cardiovascular disease,^11^ T2DM^12^ and all-cause mortality.^13^ However, there is limited data exploring the association between PA at different intensity levels and abdominal adiposity. In addition, studies using objective assessment of PA and body composition are scarce and limited in terms of their cross-sectional design^14^ and focus on sedentary time (SED-time), whisch is defined as time spent watching television or as no participation in regular sports or strenuous PA.^14,15^ Finally, although several cohort studies have examined the bidirectionality of this relationship and demonstrated that higher body mass index (BMI) or body weight is associated with future physical inactivity^16,17^ or longer time spent sedentary,^15,18, 19, 20^ none have confirmed that physical inactivity has long-term appreciable effects on the development of obesity or increases in BMI.

A better understanding of the magnitude and direction of the association between objectively measured components of PA and BF in high-risk groups is needed to develop tailored PA interventions. Using data from the ProActive study, we aimed to examine the association between objectively measured moderate-to-vigorous PA (MVPA), SED-time, and total and abdominal BF at three time points (baseline, 1 year and 7 years later). The possible bidirectionality of these associations was also assessed.

## Methods

### Study design and population

ProActive is a randomised controlled trial of the efficacy of an evidence-, family- and theory-based intervention programme to increase PA among individuals at high risk of T2DM, defined as having a parental history of T2DM.^21^ Full details of the study have been described elsewhere.^21^ In brief, the offspring of T2DM patients, identified from either diabetes registers or family history medical records in 20 UK general practices, were asked to complete a short screening questionnaire to exclude very active individuals.^22,23^ Of the 465 eligible individuals, 365 were randomly assigned to one of the following three interventions^24^: brief written advice (control group) or a behavioural change programme at two levels of intensity delivered either by telephone (distance) or face-to-face in the family home. As the main trial results showed no significant effect on 1-year change in objectively measured daytime PA,^24^ the trial arms were pooled and a cohort analysis was conducted. Participants with complete data on objectively measured PA (baseline: *n*=240, 1 year: *n*=233 and 7 years: *n*=250) were considered for this analysis. Those with missing data on BF parameters were excluded (*n*=9 at baseline, *n*=11 at 1 year and *n*=19 at 7 years). One participant became a wheel-chair user at 7 years and was excluded. Therefore, the final sample comprised *n*=231 at baseline, *n*=222 at 1 year and *n*=230 at 7 years. All participants gave written informed consent. Ethical approval was provided by the Cambridge Central REC (reference number 09/110308/3), and the Declaration of Helsinki was adhered to during all phases of the study. This trial is registered as ISRCTN 61323766.

### Assessment of PA

Free-living PA was measured with accelerometry (MTI Actigraph, Manufacturing Technology, Fort Walton Beach, FL, USA) over four consecutive days at baseline and follow-up time points, as previously described.^25^ The Actigraph model WAM 7164 (ActiGraph LLC, Pensacola, FL, USA) was used at baseline and 1-year follow-up, and model GT1M at 7-year follow-up. Both models provide an integrated measure of acceleration and movement frequency,^26, 27, 28^ and a correction factor of 0.91 was used to achieve comparability between the models (model 7164=GT1M/0.91).^29^ Participants wore the accelerometer on the lower back during waking hours, except for water-based activities. Non-wear time was defined as continuous strings of zero lasting for >60 min. Participants were excluded if their monitor wear time did not exceed 500 min per day for at least 3 days (*n*=41 at all measurement time points). Volume of activity was considered to represent total PA and was calculated as total accumulated activity counts divided by total wear time, expressed in counts per minute. Average daily time spent sedentary, in light-intensity PA and in MVPA were derived using <100 counts min^−1^ (refs 25,30), 100–1951 and ⩾1952 counts min^−1^ (ref. 31) cutoffs to summarise the movement intensity time-series records, respectively. A customised programme was used for data cleaning and reduction (MAHUffe, available from www.mrc-epid.cam.ac.uk/resources).

### Anthropometric measures

Body weight, height, hip and waist circumference (WC) were measured according to identical standard operating procedures at all time points. BMI was calculated as weight in kilograms divided by the square of height in meters. Resistance was assessed using a standard bio-impedance technique (Bodystat; Isle of Man, UK) with good reliability^32^ and validity.^33^ Total body water and fat-free mass (FFM) were calculated from published equations^34^ using body weight and the impedance index (square of height divided by resistance). Fat mass (FM) was computed as the difference between body weight and FFM. BF% was calculated as FM divided by total body weight. FM index (FMI) was computed as FM in kilograms divided by the square of height in meters.

### Self-reported covariates

Smoking status was categorised as current, former or never. Age at completion of full-time education was considered as a proxy for socioeconomic status and analysed as a binary variable (below/above 16 years).

### Statistical analysis

Descriptive statistics (means and standard deviations (SD)) at baseline and follow-ups were calculated for the whole sample and for women and men separately. *T*-tests were used to examine differences by sex, and differences in baseline characteristics between those with and without follow-up data and accelerometer data. Each PA variable and fat index was regressed against follow-up time to assess the time trend for the whole sample, and for women and men separately.

The associations between MVPA and SED-time with total and abdominal BF were assessed using mixed effects regression models, including up to three values of each exposure and outcome per person. The algebraic equations used to model the associations under consideration are detailed in Supplementary Appendix 1. No significant interactions with sex were found, so results are therefore presented for both sexes combined. Two levels of adjustment were considered in the analysis. Model 1 was adjusted for age (baseline), sex, trial group arm, monitor wear time, follow-up time, smoking and socioeconomic status (baseline). To determine the trajectory of the association over time, an interaction term with follow-up time was added to Model 1, and the main effects of MVPA and SED-time on BF indices after 1 year and 7 years were calculated (Model 1a). Model 2 was adjusted for MVPA (when the exposure was SED-time) or SED-time (when the exposure was MVPA) in addition to the covariates in Model 1. When MVPA or SED-time was the outcome, FM, WC, BF% and FMI were included as exposures in separate models adjusted for confounders as described above. All exposure and outcome variables were standardised using means and SDs of baseline values. Thus, the β-coefficients represent the change in the outcome (in s.d.’s) associated with a 1 s.d. change in the exposure. This enabled direct comparisons of the effects of SED-time and MVPA on different fat indices and *vice versa* under the assumption that the variables were measured with the same degree of error. All statistical tests were two-sided with significance defined as *P*<0.05. All analyses were completed using STATA 13 (STATA, College Station, TX, USA).

## Results

### Participant characteristics

Participant characteristics at baseline and follow-ups are summarised in Table 1 for both sexes combined and separately in men and women. Median (interquartile range (IQR)) follow-up time was 7.4 (7.1–7.7) years. There was a significant increase in weight, BMI and fat indices over time in both sexes. Men had significantly higher weight, FFM, WC and lower FM, BF% and FMI than women at all time points. In both sexes, average time in MVPA was below 30 min per day at all time points, and 70%, 64% and 65% of participants did not meet current PA recommendations at baseline, 1-year and 7-year follow-up, respectively.^3^ Compared with women, men spent substantially more time in both MVPA and sedentary. Pearson’s correlation coefficients between time spent in MVPA and sedentary were as follows: *r*=−0.14 (*P*=0.036), *r*=−0.18 (*P*=0.007) and *r*=−0.17 (*P*=0.006) for baseline, 1-year and 7-year follow-up, respectively, whereas the corresponding correlations between time in light-intensity PA and MVPA were *r*=0.09, *r*=0.24 and *r*=0.10 (all *P*>0.05). There was a significant negative correlation between time in light-intensity PA and sedentary at all time points: *r*=−0.64, *r*=−0.64 and *r*=−0.57 for baseline, 1-year and 7-year follow-up, respectively (all *P*<0.001). The volume of activity (counts per minute) and time in MVPA were strongly and positively correlated (*r*>0.8; *P*<0.001) at all time points. Mean (s.d.) SED-time increased in both sexes from 8.1 (1.5) h per day at baseline to 9.0 (1.7) h per day at the 7-year follow-up (*P*<0.01). The mean proportion of wear time spent in MVPA was between 3 and 4% at all time points in both sexes, whereas the mean proportion spent sedentary ranged from 58 to 61% in women and from 60 to 65% in men. Mean (s.d.) time at light-intensity PA at baseline was 5.3 (1.2) h per day in the whole sample that remained consistent over time and did not differ between the sexes.

There were no significant differences in BF indices at baseline between individuals with and without sufficient accelerometer information (data not shown). Participants with and without complete follow-up data on PA and body composition were comparable for all baseline characteristics, except for sex, with more women with incomplete follow-up data (data not shown).

### Mixed effects regression models with BF indicators as outcomes

Table 2 shows the estimated associations of MVPA and SED-time (exposures) with abdominal and total BF indices (outcomes) from the mixed effects model. MVPA was inversely associated with all adiposity indices, after adjustment for age, sex, intervention arm, monitor wear time, follow-up time, smoking status and socioeconomic status (Model 1). Standardised β-coefficients for MVPA ranged from −0.07 (95% confidence interval (CI): −0.10, −0.03) for body weight to −0.13 (95% CI: −0.18, −0.08) for BF%, indicating an inverse association with all outcomes. For weight, WC and FMI the size of the interaction between MVPA and follow-up time was small (Model 1a) but statistically significant, suggesting that the inverse association between MVPA and BF strengthened over time. For example, the main effect (β (95% CI)) of MVPA on body weight at 1 year was −0.03 (−0.08, 0.02), and reached −0.08 (−0.11, −0.04) at 7 years. This corresponds to a reduction in body weight of 0.5 kg (95% CI: 0.2, 1.4) associated with 16.8 min per day increase in MVPA at 1 year, and a reduction in body weight of 1.4 kg (95% CI: 0.7, 1.9) associated with that same increase in MVPA at 7 years. The corresponding reductions in fat mass associated with the same amount of MVPA were 0.67 kg (95% CI: 0.03, 1.33) at 1 year and 1.2 kg (95% CI: 0.7, 1.7) at 7 years. Furthermore, the overall effect of MVPA on BF indices was robust to adjustment for SED-time, indicating independent associations with all adiposity indicators (Model 2). A sensitivity analysis with light-intensity PA as exposure (Models 1 and 2 adjusted for MVPA instead of SED-time) yielded associations of similar magnitude and the same direction as those observed for MVPA, and there was no interaction with follow-up time.

SED-time was significantly positively associated with all fat indices except WC, with standardised β-coefficients ranging from 0.11 (95% CI: 0.06, 0.16) for body weight to 0.14 (95% CI: 0.08, 0.21) for FM (Model 1; Table 2). Interactions between SED-time and follow-up time (Model 1a) were not statistically significant, so there was no evidence that the effect of SED-time on the outcomes changed over time. After additional adjustment for MVPA (Model 2), the main effect of SED-time on BF indices was somewhat attenuated but remained statistically significant (except for BF%), with the greatest attenuation (30%) where FMI was the outcome. On the original scale, every additional 1.5 h per day spent sedentary was associated with a 1.5 kg (95% CI: 0.7, 2.4) increase in body weight independent of MVPA.

### Mixed effects regression models with MVPA or SED-time as outcomes

When MVPA was modelled as the outcome (Table 3), inverse independent associations were consistently observed for all BF indices, with an approximately threefold greater magnitude compared with the relationships where MVPA was considered the exposure variable (Table 2). Adjusted standardised β-coefficients ranged from *β*=−0.25 (95% CI: −0.36, −0.15) for WC to *β*=−0.30 (95% CI: −0.41, −0.19) for BF%. Positive associations were found between all adiposity indices and SED-time as the outcome (Table 3), and were of a similar magnitude as those observed in the models where SED-time was the exposure (Table 2). No significant interactions between the BF indices and follow-up time were identified. In a sensitivity analysis with light-intensity PA as outcome, standardised β-coefficients were of opposite direction and the same magnitude as compared with those observed for SED-time.

## Discussion

This study used repeated measures over a 7-year period to assess the associations between objectively measured time spent in MVPA and sedentary with total and abdominal BF in middle-aged adults with parental history of T2DM. We also assessed the extent to which these associations changed over time and the bidirectionality of the associations. Overall, our findings suggest that MVPA and SED-time have substantial and independent effects on both total and abdominal BF. A 1.5-h reduction in SED-time and a 16-min increase in MVPA per day were associated with a 1.4-kg and 0.5-kg reduction in body weight over 1 year, respectively. There is evidence that MVPA exerts stronger beneficial effects on BF over time, which was not the case for SED-time, whose detrimental effect did not vary significantly over time. There was a significant inverse association between all fat indicators and MVPA (outcome) that did not appear to vary over time. This association was of approximately threefold greater magnitude compared with the relationship of MVPA (exposure) and BF (outcome). Similarly, we found a significant positive association between all BF indices and SED-time, and this relationship remained stable over the follow-up. The effects of BF seem to be stronger on MVPA than on SED-time. Taken together, our analyses demonstrate bidirectional associations of PA and sedentary behaviour with BF. Relative magnitude of the association between BF and MVPA is greater than the reciprocal association, thereby highlighting the importance of considering BF as a determinant of decreasing activity as well a potential consequence.

To the best of our knowledge, no previous studies have examined the associations between MVPA, SED-time and using objective methods to repeatedly assess exposure and outcome in a population with a parental history of T2DM. A systematic review of prospective cohort studies using objective assessment of PA among children, adolescents and adults has shown that total PA (expressed as counts per minute or as energy expenditure) is not a predictor of weight gain.^35^ Among other studies that used objective assessments of PA and body composition, Healy *et al.*^14,30^ found a positive cross-sectional association between SED-time and WC in the United States and Australian general population samples. Ekelund *et al.*,^15^ on the other hand, did not detect any significant role of SED-time for predicting changes in adiposity. The latter three studies, however, did not examine individuals at high risk of T2DM and focused only on SED-time as the exposure.

MVPA predicted WC in our sample, which is in keeping with the results from a few large-scale epidemiological studies using total PA.^36, 37, 38^ Although these studies used large sample sizes and have a long follow-up, they are limited by their use of self-reported PA measures, which might have led to misclassification bias. Results from the EPIC-PANACEA study indicate that low total PA predicts a gain in abdominal adiposity in the general adult population from 10 European countries (*N*>280 000, age range 25–79 years at baseline).^36^ Using a similar analytical approach as the current analyses, May *et al.*^38^ demonstrated that inverse prospective associations of total PA with gains in weight and WC in 4944 Dutch adults followed-up for 10 years, with three follow-up assessments of PA, weight and WC. Hamer *et al.*^39^ have shown that an increase in MVPA of 2.5 h per week or more between two follow-ups (5 years apart) was associated with a reduction in BMI and WC compared with stable MVPA over the same period in the Whitehall II cohort. However, findings from the Women’s Health Study showed an interaction between BMI and PA on weight gain, so that the significant inverse relationship between PA and weight gain existed only among those with BMI <25 kg m^−^^2^ but not in overweight or obese participants.^37^

Our observation that increased BF is associated with reduced MVPA and increased SED-time is biologically plausible and can be explained by physiological changes such as dyspnea and musculoskeletal problems that may lead to discomfort and subsequent drop in PA of this intensity. These results are consistent with the findings from the EPIC-Norfolk cohort,^16^ showing that greater weight gain over time is associated with physical inactivity at follow-up, as well as results from the Copenhagen City Heart Study, indicating that higher BMI predicts physical inactivity.^17^ Other studies provided evidence that greater baseline BMI is related to later increase in SED-time based on self-report.^18, 19, 20^ Only a few studies have assessed the bidirectional character of the relationship in question and used objective methods to measure PA. Results from the Ely Study^15^ suggest that greater body weight, FM and BMI predict higher levels of SED-time as assessed by individually calibrated heart rate monitoring. However, the Early Bird study involving children born in 1995/96 has shown that MVPA is associated with improvements in the composite metabolic risk score, but not with a reduction in BMI or fatness.^40^ A study^41^ involving 785 Danish children (aged 8–11 years) demonstrated that adiposity is statistically a better predictor of PA and SED-time than PA predicting adiposity. Although our study has some similarities with the Danish study in that both represent a cohort analysis of a randomised trial, differences in age, follow-up and accelerometer wearing protocols should be considered when making comparisons. Likewise, we found significant positive associations between BF and SED-time. The observed bidirectional nature of the associations highlights the need to consider inactivity not only as a determinant but also as a possible result of weight gain.

The observation that follow-up time significantly modifies the association of MVPA, but not SED-time with BF indicators may have several explanations. It is possible that an increase in MVPA over time leads to a decrease in BF, with the association becoming stronger over time because of the cumulative effects of PA. In addition, it might be that those who increase their MVPA are more health conscious and also tend to adopt a healthier diet, thereby leading to additional beneficial effects on body composition.

An important strength of our study is the use of objective methods to assess MVPA, SED-time and body composition in a well-characterised population, thereby reducing the random measurement error and recall bias associated with self-reported PA. In addition, exposures and outcomes were measured in a standardised fashion by trained staff at baseline and follow-ups, which also contributed to the reduction in the random measurement error. Furthermore, all exposures and outcomes of interest were analysed in their continuous form, which decreased the likelihood of the loss of statistical power that would have occurred had the categorical variables been used. Mixed effects models were performed using exposure and outcome data at all time points that optimised statistical power and provided a framework to take into account both the within-individual and between-individual variance. The participants were followed-up for a median of 7.4 years that allowed us to assess medium- to long-term effects of MVPA and SED-time on total and abdominal BF and *vice versa*. Moreover, we examined the interactions with time and were able to quantify the exact increase in BF indices associated with the 1 s.d. (16.8 min per day) increase in MVPA at each time point that provides an important public health message.

Nevertheless, several limitations need to be taken into account in the interpretation of our results. First, the relatively small population sample of middle-aged Caucasians at high risk for T2DM may limit the generalisability of our findings to other populations. Second, although we controlled for a range of potential confounders including age, sex, cigarette smoking, socioeconomic status, as well as monitor wear time and follow-up time, residual confounding cannot be ruled out. In addition to energy expenditure, energy intake is the most important factor in determining energy balance. Dietary intake is therefore a possible confounder in the association between MVPA, SED-time and BF, which we were unable to include. Third, as participants were instructed to wear the accelerometer for 4 days only, the observed levels of PA may not be completely representative of their usual PA. Fourth, it is possible that the validity of accelerometry for capturing activity at various intensity levels differs by obesity status through differential participation in different types of activity,^42,43^ which may introduce bias or dilute the associations. Although MVPA was assessed objectively, it is likely that the measurement of MVPA over a snapshot of 4 days was less precise than the measurement of BF. The observed greater magnitude and wider CIs of the association in which MVPA is the outcome compared with that in which MVPA is the exposure^44^ is likely stemming from differential measurement error in MVPA and adiposity variables, the latter being measured with greatest relative precision; as reported by Hutcheon *et al.,*^45^ relatively larger error in the exposure tends to attenuate an association, whereas error in the outcome increases the standard error of an effect estimate.

Our findings indicate that an increase in 16.8 min per day of MVPA would result in a decrease in FM of 1.2 kg (95% CI: 0.6, 1.7) over 7 years. On the other hand, an increase in FM of 10 kg over 7 years would result in a reduction of 4.8 min per day (95% CI: 3.1, 6.5) in MVPA, and an increase of 0.2 h per day (95% CI: 0.1, 0.3) in SED-time. Our results corroborate considerable beneficial effects of MVPA and the detrimental effects of SED-time on BF, which suggests that promoting an increase in MVPA as well as a reduction SED-time would be favourable in people with parental family history of T2DM. Our findings also show that individuals with increased BF are more likely to reduce their activity in the future that may have detrimental effects on metabolic risk. Therefore, individuals with high BF require particular attention for the promotion of MVPA and decreasing sedentary behaviour in order to avoid self-promoting weight gain^46^ and to mitigate unfavourable metabolic changes because of increased BF. Our subsidiary analyses have indicated a beneficial effect of light-intensity PA on BF. Given that adults spend a large proportion of non-sedentary awake time at light-intensity, a detailed exploration of the role that light-intensity PA may have in the accumulation of BF and its association with other cardio-metabolic outcomes across a wide range of population subgroups is warranted in future studies.

In conclusion, our results indicate that the association of MVPA and SED-time with total and abdominal BF is bidirectional among individuals at high risk for T2DM. The effects of detrimental BF profile on the reduction of MVPA seem to be stronger than the effects of increasing MVPA on a reduction in BF indices, which suggests that BF should be regarded as a determinant of MVPA. Given the independent effects of MVPA and sedentary behaviour, promoting both an increase in MVPA and a decrease in sedentary behaviour may reduce total and abdominal BF. Considering the evidence that the beneficial effects of MVPA become stronger over time, increasing MVPA may have a greater effect than reducing sedentary behaviour on BF. Further research is warranted to clarify the effects of PA (including light-intensity activities) on BF in more diverse populations.

## Figures and Tables

**Table 1:** Participant characteristics at baseline, 1-year and 7-year follow-up in the ProActive trial cohort

*Both sexes*	*Baseline*	*1-year follow-up*	*7-year follow-up*
*N*	231	222	230
Age (years)	41.3 (6.4)	42.6 (6.3)	48.7 (6.2)^***^
Weight (kg)	79.5 (17.0)	80.8 (16.8)	83.3 (17.5)*
BMI (kg m^−^^2^)	27.7 (4.9)	28.0 (4.9)	28.7 (5.1)*
Fat mass (kg)	25.1 (9.5)	25.3 (9.4)	26.4 (10.1)*
Fat-free mass (kg)	54.5 (11.6)	55.5 (11.7)	56.9 (12.6)*
WC (cm)	93.0 (13.4)	94.2 (13.2)	94.3 (13.9)^**^
BF%	31.0 (7.7)	30.9 (7.6)	31.3 (8.4)*
FMI (kg m^−^^2^)	8.9 (3.5)	8.9 (3.5)	9.2 (3.8)*
MVPA (min per day)	25.7 (16.8)	29.4 (19.4)	29.1 (21.3)
LPA (h per day)	5.2 (1.2)	5.1 (1.3)	4.8 (1.2)
Sedentary (h per day)	8.1 (1.5)	7.9 (1.6)	9.0 (1.7)^**^
Volume of activity (counts per min)	323.2 (114.6)	342.5 (125.3)	338.0 (136.4)
Monitor wear time (h per day)	13.7 (1.2)	13.5 (1.3)	14.4 (1.4)*
			
*Women*	*Baseline*	*1-year follow-up*	*7-year follow-up*
*N*	139	126	130
Age (years)	41.3 (6.3)	42.9 (6.2)	48.9 (6.3)^***^
Weight (kg)	73.1 (14.7)^†††^	73.6 (14.1)^†††^	76.2 (15.2)^***,†††^
BMI (kg m^−2^)	27.4 (5.2)	27.7(5.1)	28.5 (5.5)^***^
Fat mass (kg)	26.1 (10.2)^†^	26.3(9.9)	27.9 (10.8)^**,††^
Fat-free mass (kg)	47.0 (5.4)^†††^	47.2 (5.4)^†††^	48.3 (6.2)^†††^
WC (cm)	87.8 (12.0)^†††^	89.0 (12.0)^†††^	89.4 (13.3)^**,†††^
BF%	35.5 (7.0) ^†††^	34.7 (6.2)^†††^	35.5 (7.4)^**,†††^
FMI (kg m^−^^2^)	9.8 (3.8) ^†††^	9.9 (3.7)^†††^	10.5 (4.0)^**,†††^
MVPA (min per day)	23.6 (16.7)^***^	28.2 (20.5)	24.7 (19.1)^†††^
LPA (h per day)	5.3 (1.2)	5.1 (1.2)	5.0 (1.1)
Sedentary (h per day)	7.8 (1.4)^††^	7.8 (1.5)	8.6 (1.6)^***,†††^
Volume of activity (counts per min)	321.4 (117.3)	340.1 (122.8)	326.3 (130.3)*
Monitor wear time (h per day)	13.5 (1.2)^†††^	13.3 (1.3)^†^	14.0 (1.4) ^*,†††^
			
*Men*	*Baseline*	*1-year follow-up*	*7-year follow-up*
*N*	92	96	100
Age (years)	41.2 (6.4)	42.2 (6.3)	48.4 (6.0)^***^
Weight (kg)	89.3 (15.7)	90.2 (15.4)	92.4 (15.9)^***^
BMI (kg m^−2^)	28.3 (4.5)	28.4 (4.5)	29.0 (4.5)^***^
Fat mass (kg)	23.5 (8.3)	23.9 (8.4)	24.9 (8.9)
Fat-free mass (kg)	65.8 (9.0)	66.3 (8.4)	68.1 (9.6)
WC (cm)	100.8 (11.6)	101.0 (11.4)	100.7 (12.2)
BF%	25.6 (5.3)	25.9 (5.2)	25.7 (6.1)
FMI (kg m^−2^)	7.5 (2.6)	7.5 (2.6)	7.6 (2.8)
MVPA (min per day)	29.0 (16.6)	30.9 (17.8)	34.7 (22.8)
LPA (h per day)	5.1 (1.3)	5.1 (1.3)	4.7 (1.3)
Sedentary (h per day)	8.4 (1.4)	8.1 (1.6)	9.6 (1.7)^**^
Volume of activity (counts per min)	325.9 (111.0)	345.7 (129.0)	356.2 (143.2)
Monitor wear time (h)	14.0 (1.1)	13.7 (1.3)	14.8 (1.3)*

Abbreviations: BF%, percentage of body fat; BMI, body mass index; FMI, fat mass index (fat mass per height^2^);

LPA, light-intensity physical activity; MVPA, time spent in moderate-to-vigorous physical activity; WC, waist circumference.

Results are mean (s.d.).

*N* is the number of participants with complete data on all variables in Table 1.

*P<0.05, **P<0.01, ***P<0.001 time trend in the variable of interest (regression against follow-up time).

^†^*P*<0.05, ^††^*P*<0.01, ^†††^*P*<0.001 for the difference between men and women at each time point (*t*-test).

**Table 2:** Associations between time spent in MVPA and sedentary with fat indicators obtained from mixed effects models using data at all time points in the ProActive trial cohort

*Exposure*	*Outcome*	*Overall main effect (Model 1)*	*Interaction effect with follow-up time (Model 1a)*	*Main effect at 1 year (Model 1a)*	*Main effect at 7 years (Model 1a)*	*Overall main effect (Model 2)*
MVPA	Weight	−0.07 (−0.10, −0.03)***	−0.0074 (−0.0145, −0.0003)*	−0.03 (−0.08, 0.02)	−0.08 (−0.11, −0.04)***	−0.05 (−0.08, −0.01)**
	WC	−0.08 (−0.13, −0.04)***	−0.013 (−0.022, −0.003)**	−0.02 (−0.08, 0.05)	−0.10 (−0.14, −0.05)***	−0.07 (−0.12, −0.02)**
	FM	−0.12 (−0.16, −0.07)***	−0.009 (−0.019, 0.001)	−0.072 (−0.141, −0.003)*	−0.13 (−0.18, −0.08)***	−0.09 (−0.14, −0.04)***
	BF%	−0.13 (-0.18, −0.08)***	−0.009 (−0.020, 0.003)	−0.09 (−0.16, −0.01)**	−0.14 (−0.19, −0.09)***	−0.11 (−0.17, −0.06)***
	FMI	−0.11 (-0.16, −0.06)***	−0.010 (−0.020, −0.001)*	−0.06 (−0.13, 0.01)	−0.12 (−0.17, −0.07)***	−0.08 (−0.13, −0.04) **
						
Sedentary time	Weight	0.11 (0.06, 0.16)***	−0.001 (−0.008, 0.007)	0.11 (0.05, 0.17)***	0.10 (0.06, 0.16)***	0.09 (0.04, 0.14)**
	WC	0.06 (−0.01, 0.12)	0.003 (−0.009, 0.010)	0.06 (−0.01, 0.14)	0.06 (−0.01, 0.13)	0.03 (−0.04, 0.09)
	FM	0.14 (0.08, 0.21)***	0.003 (−0.008, 0.014)	0.13 (0.05, 0.21)**	0.15 (0.08, 0.22)***	0.10 (0.03, 0.17)**
	BF%	0.11 (0.04, 0.18)**	0.004 (−0.008, 0.016)	0.09 (0.01, 0.18)*	0.12 (0.05, 0.19)**	0.06 (−0.01, 0.13)
	FMI	0.13 (0.06, 0.19)***	0.004 (−0.007, 0.014)	0.11 (0.03, 0.19)*	0.13 (0.07, 0.20)**	0.09 (0.02, 0.16)*

Abbreviations: BF%, percentage of body fat; CI, confidence interval; FM, fat mass; FMI, fat mass index (fat mass per height

^2^); MVPA, time spent in moderate-to-vigorous physical activity; WC, waist circumference.

Results are standardised regression coefficients (95% CI), that is, the change in outcome (in s.d. units) per 1 s.d. higher value of the exposure.

Model 1 adjusted for age, sex, intervention arm, monitor wear time, follow-up time, smoking status and socioeconomic status.

Model 1a: Model 1+ interaction term between exposure (MVPA or sedentary time) and follow-up time.

Model 2: Model 1+ sedentary time/MVPA.

*P<0.05, **P<0.01, ***P<0.001.

Data for education and/or smoking were missing for 37 participants who were therefore excluded.

**Table 3:** Associations between fat indicators and time spent in MVPA and sedentary obtained from mixed effects models using data at all time points in the ProActive trial cohort

*Outcome*	*Exposure*	*Overall main effect (Model 1)*	*Interaction effect with follow-up time (Model 1a)*	*Main effect at 1 year (Model 1a)*	*Main effect at 7 year (Model 1a)*
MVPA	Weight	−0.29 (−0.39; −0.18)***	−0.01 (−0.03, 0.01)	−0.25 (−0.38; −0.12)***	−0.31 (−0.43; −0.19)***
	WC	−0.25 (−0.36; −0.15)***	−0.01 (−0.03, 0.01)	−0.21 (−0.33; −0.08)**	−0.28 (−0.40; −0.17)***
	FM	−0.27 (−0.36; −0.18)***	−0.01 (−0.04, 0.01)	−0.22 (−0.34; −0.09)***	−0.31 (−0.41; −0.20)***
	BF%	−0.30 (−0.41; −0.19)***	−0.01 (−0.03, 0.01)	−0.25 (−0.38; −0.11)***	−0.33 (−0.44; −0.21)***
	FMI	−0.27 (−0.36; −0.17)***	−0.01 (−0.03, 0.01)	−0.22 (−0.35; −0.10)***	−0.29 (−0.40; −0.18)***
					
Sedentary time	Weight	0.16 (0.07; 0.25)***	0.001 (−0.014, 0.016)	0.16 (0.06; 0.27)***	0.17 (0.07; 0.26)***
	WC	0.10 (0.02; 0.18)**	0.003 (−0.012, 0.018)	0.09 (0.01; 0.19)	0.11 (0.02; 0.20)*
	FM	0.15 (0.07; 0.22)***	0.003 (−0.011, 0.018)	0.13 (0.04; 0.23)**	0.15 (0.07; 0.24)***
	BF%	0.14 (0.05; 0.22)**	0.005 (−0.010, 0.019)	0.12 (0.01; 0.22)*	0.15 (0.06; 0.24)**
	FMI	0.14 (0.06; 0.22)**	0.003 (−0.012, 0.018)	0.13 (0.03; 0.24)**	0.15 (0.06; 0.23)**

Abbreviations: BF%, percentage of body fat; CI, confidence interval; FM, fat mass; FMI, fat mass index (fat mass per height

^2^); MVPA, time spent in moderate-to-vigorous physical activity; WC, waist circumference.

Results are standardised regression coefficients (95% CI), following standardisation of exposure and outcome variables, that is, the change in outcome (in s.d. units) per 1 s.d. higher value of the exposure.

Each fat indicator was included in a separate model as exposure.

Analyses were adjusted for age, sex, intervention arm, monitor wear time, follow-up time, smoking status and socioeconomic status.

*P<0.05, **P<0.01, ***P<0.001.

Data for education and/or smoking were missing for 37 participants who were therefore excluded.

## References

[bib1] World Health Organization. Global Health Risks: Mortality and Burden of Disease Attributable to Selected Major Risks. WHO Press: Geneva, Switzerland, 2009.

[bib2] Harrison TA, Hindorff LA, Kim H, Wines RC, Bowen DJ, McGrath BB et al. Family history of diabetes as a potential public health tool. Am J Prev Med 2003; 24: 152–159. 12568821 10.1016/s0749-3797(02)00588-3

[bib3] Haskell WL, Lee IM, Pate RR, Powell KE, Blair SN, Franklin BA et al. Physical activity and public health: updated recommendation for adults from the American College of Sports Medicine and the American Heart Association. Circulation 2007; 116: 1081–1093. 17671237 10.1161/CIRCULATIONAHA.107.185649

[bib4] Sargeant LA, Wareham NJ, Khaw KT. Family history of diabetes identifies a group at increased risk for the metabolic consequences of obesity and physical inactivity in EPIC-Norfolk: a population-based study. The European Prospective Investigation into Cancer. Int J Obes Relat Metab Disord 2000; 24: 1333–1339. 11093296 10.1038/sj.ijo.0801383

[bib5] Hu FB, Li TY, Colditz GA, Willett WC, Manson JE. Television watching and other sedentary behaviors in relation to risk of obesity and type 2 diabetes mellitus in women. JAMA 2003; 289: 1785–1791. 12684356 10.1001/jama.289.14.1785

[bib6] Raynor DA, Phelan S, Hill JO, Wing RR. Television viewing and long-term weight maintenance: results from the National Weight Control Registry. Obesity (Silver Spring) 2006; 14: 1816–1824. 17062812 10.1038/oby.2006.209

[bib7] Warren TY, Barry V, Hooker SP, Sui X, Church TS, Blair SN. Sedentary behaviors increase risk of cardiovascular disease mortality in men. Med Sci Sports Exerc 2010; 42: 879–885. 19996993 10.1249/MSS.0b013e3181c3aa7e PMC2857522

[bib8] Krishnan S, Rosenberg L, Palmer JR. Physical activity and television watching in relation to risk of type 2 diabetes: the Black Women’s Health Study. Am J Epidemiol 2009; 169: 428–434. 19056835 10.1093/aje/kwn344 PMC2726641

[bib9] Dunstan DW, Salmon J, Healy GN, Shaw JE, Jolley D, Zimmet PZ et al. Association of television viewing with fasting and 2-h postchallenge plasma glucose levels in adults without diagnosed diabetes. Diabetes Care 2007; 30: 516–522. 17327314 10.2337/dc06-1996

[bib10] Wareham NJ, van Sluijs EM, Ekelund U. Physical activity and obesity prevention: a review of the current evidence. Proc Nutr Soc 2005; 64: 229–247. 15960868 10.1079/pns2005423

[bib11] Lissner L, Bjorkelund C, Heitmann BL, Seidell JC, Bengtsson C. Larger hip circumference independently predicts health and longevity in a Swedish female cohort. Obes Res 2001; 9: 644–646. 11595782 10.1038/oby.2001.85

[bib12] Carey VJ, Walters EE, Colditz GA, Solomon CG, Willett WC, Rosner BA et al. Body fat distribution and risk of non-insulin-dependent diabetes mellitus in women. The Nurses’ Health Study. Am J Epidemiol 1997; 145: 614–619. 9098178 10.1093/oxfordjournals.aje.a009158

[bib13] Bigaard J, Tjonneland A, Thomsen BL, Overvad K, Heitmann BL, Sorensen TI. Waist circumference, BMI, smoking, and mortality in middle-aged men and women. Obes Res 2003; 11: 895–903. 12855760 10.1038/oby.2003.123

[bib14] Healy GN, Matthews CE, Dunstan DW, Winkler EA, Owen N. Sedentary time and cardio-metabolic biomarkers in US adults: NHANES 2003-06. European heart journal 2011; 32: 590–597. 21224291 10.1093/eurheartj/ehq451 PMC3634159

[bib15] Ekelund U, Brage S, Besson H, Sharp S, Wareham NJ. Time spent being sedentary and weight gain in healthy adults: reverse or bidirectional causality? Am J Clin Nutr 2008; 88: 612–617. 18779275 10.1093/ajcn/88.3.612

[bib16] Hutcheon JA, Zhang X, Cnattingius S, Kramer MS, Platt RW. Customised birthweight percentiles: does adjusting for maternal characteristics matter? BJOG 2008; 115: 1397–1404. 18823489 10.1111/j.1471-0528.2008.01870.x

[bib17] Petersen L, Schnohr P, Sorensen TI. Longitudinal study of the long-term relation between physical activity and obesity in adults. Int J Obes Relat Metab Disord 2004; 28: 105–112. 14647181 10.1038/sj.ijo.0802548

[bib18] Bak H, Petersen L, Sorensen TI. Physical activity in relation to development and maintenance of obesity in men with and without juvenile onset obesity. Int J Obes Relat Metab Disord 2004; 28: 99–104. 14610530 10.1038/sj.ijo.0802525

[bib19] Mortensen LH, Siegler IC, Barefoot JC, Gronbaek M, Sorensen TI. Prospective associations between sedentary lifestyle and BMI in midlife. Obesity (Silver Spring) 2006; 14: 1462–1471. 16988090 10.1038/oby.2006.166

[bib20] Pulsford RM, Stamatakis E, Britton AR, Brunner EJ, Hillsdon MM. Sitting behavior and obesity: evidence from the Whitehall II study. Am J Prev Med 2013; 44: 132–138. 23332328 10.1016/j.amepre.2012.10.009 PMC3550520

[bib21] Williams K, Prevost AT, Griffin S, Hardeman W, Hollingworth W, Spiegelhalter D et al. The ProActive trial protocol—a randomised controlled trial of the efficacy of a family-based, domiciliary intervention programme to increase physical activity among individuals at high risk of diabetes [ISRCTN61323766]. BMC Public Health 2004; 4: 48. 15491494 10.1186/1471-2458-4-48 PMC526256

[bib22] Godin G, Shephard RJ. A simple method to assess exercise behavior in the community. Can J Appl Sport Sci 1985; 10: 141–146. 4053261

[bib23] Wareham NJ, Jakes RW, Rennie KL, Schuit J, Mitchell J, Hennings S et al. Validity and repeatability of a simple index derived from the short physical activity questionnaire used in the European Prospective Investigation into Cancer and Nutrition (EPIC) study. Public Health Nutr 2003; 6: 407–413. 12795830 10.1079/PHN2002439

[bib24] Kinmonth AL, Wareham NJ, Hardeman W, Sutton S, Prevost AT, Fanshawe T et al. Efficacy of a theory-based behavioural intervention to increase physical activity in an at-risk group in primary care (ProActive UK): a randomised trial. Lancet 2008; 371: 41–48. 18177774 10.1016/S0140-6736(08)60070-7

[bib25] Ekelund U, Griffin SJ, Wareham NJ. Physical activity and metabolic risk in individuals with a family history of type 2 diabetes. Diabetes Care 2007; 30: 337–342. 17259504 10.2337/dc06-1883

[bib26] John D, Tyo B, Bassett DR. Comparison of four ActiGraph accelerometers during walking and running. Med Sci Sports Exerc 2010; 42: 368–374. 19927022 10.1249/MSS.0b013e3181b3af49 PMC2809132

[bib27] Kozey SL, Lyden K, Howe CA, Staudenmayer JW, Freedson PS. Accelerometer output and MET values of common physical activities. Med Sci Sports Exerc 2010; 42: 1776–1784. 20142781 10.1249/MSS.0b013e3181d479f2 PMC2924952

[bib28] Ried-Larsen M, Brond JC, Brage S, Hansen BH, Grydeland M, Andersen LB et al. Mechanical and free living comparisons of four generations of the Actigraph activity monitor. Int J Behav Nutr Phys Act 2012; 9: 113. 22971175 10.1186/1479-5868-9-113 PMC3463450

[bib29] Corder K, Brage S, Ramachandran A, Snehalatha C, Wareham N, Ekelund U. Comparison of two Actigraph models for assessing free-living physical activity in Indian adolescents. J Sports Sci 2007; 25: 1607–1611. 17852668 10.1080/02640410701283841

[bib30] Healy GN, Wijndaele K, Dunstan DW, Shaw JE, Salmon J, Zimmet PZ et al. Objectively measured sedentary time, physical activity, and metabolic risk: the Australian Diabetes, Obesity and Lifestyle Study (AusDiab). Diabetes Care 2008; 31: 369–371. 18000181 10.2337/dc07-1795

[bib31] Freedson PS, Melanson E, Sirard J. Calibration of the Computer Science and Applications, Inc. accelerometer. Med Sci Sports Exerc 1998; 30: 777–781. 9588623 10.1097/00005768-199805000-00021

[bib32] Shanholtzer BA, Patterson SM. Use of bioelectrical impedance in hydration status assessment: reliability of a new tool in psychophysiology research. Int J Psychophysiol 2003; 49: 217–226. 14507440 10.1016/s0167-8760(03)00143-0

[bib33] Simpson JA, Lobo DN, Anderson JA, Macdonald IA, Perkins AC, Neal KR et al. Body water compartment measurements: a comparison of bioelectrical impedance analysis with tritium and sodium bromide dilution techniques. Clin Nutr 2001; 20: 339–343. 11478832 10.1054/clnu.2001.0398

[bib34] Sun SS, Chumlea WC, Heymsfield SB, Lukaski HC, Schoeller D, Friedl K et al. Development of bioelectrical impedance analysis prediction equations for body composition with the use of a multicomponent model for use in epidemiologic surveys. Am J Clin Nutr 2003; 77: 331–340. 12540391 10.1093/ajcn/77.2.331

[bib35] Wilks DC, Besson H, Lindroos AK, Ekelund U. Objectively measured physical activity and obesity prevention in children, adolescents and adults: a systematic review of prospective studies. Obes Rev 2011; 12: e119–e129. 20604868 10.1111/j.1467-789X.2010.00775.x

[bib36] Ekelund U, Besson H, Luan J, May AM, Sharp SJ, Brage S et al. Physical activity and gain in abdominal adiposity and body weight: prospective cohort study in 288 498 men and women. Am J Clin Nutr 2011; 93: 826–835. 21346093 10.3945/ajcn.110.006593

[bib37] Lee IM, Djousse L, Sesso HD, Wang L, Buring JE. Physical activity and weight gain prevention. JAMA 2010; 303 : 1173–1179. 20332403 10.1001/jama.2010.312 PMC2846540

[bib38] May AM, Bueno-de-Mesquita HB, Boshuizen H, Spijkerman AM, Peeters PH, Verschuren WM. Effect of change in physical activity on body fatness over a 10-y period in the Doetinchem Cohort Study. Am J Clin Nutr 2010; 92: 491–499. 20573793 10.3945/ajcn.2010.29404

[bib39] Hamer M, Brunner EJ, Bell J, Batty GD, Shipley M, Akbaraly T et al. Physical Activity Patterns Over 10 Years in Relation to Body Mass Index and Waist Circumference: The Whitehall II Cohort Study. Obesity (Silver Spring) 2013; 21 : E755–E761. 23512753 10.1002/oby.20446

[bib40] Metcalf BS, Voss LD, Hosking J, Jeffery AN, Wilkin TJ. Physical activity at the government-recommended level and obesity-related health outcomes: a longitudinal study (Early Bird 37). Arch Dis Child 2008; 93: 772–777. 18591181 10.1136/adc.2007.135012

[bib41] Hjorth MF, Chaput J-P, Ritz C, Dalskov S-M, Andersen R, Astrup A et al. Fatness predicts decreased physical activity and increased sedentary time, but not vice versa: support from a longitudinal study in 8- to 11-year-old children. Int J Obes (Lond) 2014; e-pub ahead of print 5 December 2013; doi:10.1038/ijo.2013.229. 10.1038/ijo.2013.229 24304596

[bib42] Lyden K, Kozey SL, Staudenmeyer JW, Freedson PS. A comprehensive evaluation of commonly used accelerometer energy expenditure and MET prediction equations. Eur J Appl Physiol 2011; 111: 187–201. 20842375 10.1007/s00421-010-1639-8 PMC3432480

[bib43] Spees CK, Scott JM, Taylor CA. Differences in amounts and types of physical activity by obesity status in US adults. Am J Health Behav 2012; 36: 56–65. 22251783 10.5993/ajhb.36.1.6 PMC3888549

[bib44] Wareham N, Brage S. Commentary: Physical activity and obesity; scientific uncertainty and the art of public health messaging. Int J Epidemiol 2013; 42: 1843–1845. 24415620 10.1093/ije/dyt164

[bib45] Hutcheon JA, Chiolero A, Hanley JA. Random measurement error and regression dilution bias. BMJ 2010; 340: c2289. 20573762 10.1136/bmj.c2289

[bib46] Christiansen E, Swann A, Sorensen TI. Feedback models allowing estimation of thresholds for self-promoting body weight gain. J Theor Biol 2008; 254: 731–736. 18671981 10.1016/j.jtbi.2008.07.004

