# Supplementary material for: Physical activity, sedentary time and gain in overall and central body fat: 7-year follow-up of the ProActive trial cohort
Source: Int J Obes (Lond). 2014 May 20;39(1):142–8. doi: 10.1038/ijo.2014.66 (PMC4113455; doi:10.1038/ijo.2014.66)
Supplement: Supplementary Appendix 1 [file ijo201466x1.doc]

**Appendix 1 Algebraic equations for the regression models**

***1.Fat-indices modelled as outcomes***

**Model 1**

a) Exposure of interest MVPA

zFI_ij_ = β_0_ + u_0i_ + β_1_ x age_i0_ + β_2_ x sex + β_3_ x time_ij_ + β_4_ x zMVPA_ij_ + other covariates +ε_ij_

b) Exposure of interest SED-time

zFI_ij_ = β_0_ + u_0i_ + β_1_ x age_i0_ + β_2_ x sex + β_3_ x time_ij_ + β_4_ x zSED-time_ij_ + other covariates +ε_ij_

**Model 1a**

a) Exposure of interest MVPA

zFI_ij_ = β_0_ + u_0i_ + β_1_ x age_i0_ + β_2_ x sex + β_3_ x time_ij_ + β_4_ x zMVPA_ij_ + γ x [time_ij_ x zMVPA_ij_)] +other covariates +ε_ij_

b) Exposure of interest SED-time

zFI_ij_ = β_0_ + u_0i_ + β_1_ x age_i0_ + β_2_ x sex + β_3_ x time_ij_ + β_4_ x zSED-time_ij_ + γ x [time_ij_ x zSED-time_ij_)] +other covariates +ε_ij_

**Model 2**

zFI_ij_ = β_0_ + u_0i_ + β_1_ x age_i0_ + β_2_ x sex + β_3_ x time_ij_ + β_4_ x zMVPA_ij_ + β_5_ x zSED-time_ij_ + other covariates +ε_ij_

***2.MVPA/sedentary time modelled as outcomes***

a) Outcome of interest MVPA

**Model 1**

zMVPA_ij_ = β_0_ + u_0i_ + β_1_ x age_i0_ + β_2_ x sex + β_3_ x time_ij_ + β_4_ x zFI_ij_ + other covariates +ε_ij_

**Model 1a**

zMVPA_ij_ = β_0_ + u_0i_ + β_1_ x age_i0_ + β_2_ x sex + β_3_ x time_ij_ + β_4_ x zFI_ij_ + γ x [time_ij_ x zFI_ij_)] +other covariates +ε_ij_

b) Outcome of interest SED-time

**Model 1**

z SED-time _ij_ = β_0_ + u_0i_ + β_1_ x age_i0_ + β_2_ x sex + β_3_ x time_ij_ + β_4_ x zFI_ij_ + other covariates +ε_ij_

**Model 1a**

z SED-time _ij_ = β_0_ + u_0i_ + β_1_ x age_i0_ + β_2_ x sex + β_3_ x time_ij_ + β_4_ x zFI_ij_ + γ x [time_ij_ x zFI_ij_)] +other covariates +ε_ij_

The term *other covariates* in algebraic expressions refers to the sum of the products of a value of a particular covariate (intervention arm, monitor wear time, follow-up time, smoking status and socioeconomic status) and the corresponding β-coefficient

FI-fat indicator [in separate models- waist circumference, body weight, fat mass, fat mass index (fat mass/height^2^), percentage of body fat]; MVPA- time spent in moderate to vigorous physical activity; SED-time- sedentary time

Level 2 random effects:

u_0i_ ~ N (0, σ_0_^2^)

Level 1 residuals:

ε_ij_ ~ N (0, σ_ε_^2^)

i=individual (i=1 to 231)

j=measurement occasion (j=0,1,2)

time_ij_ = years since baseline of measurement occasion j for individual i, time_i0_ = 0

γ represents the difference in the effect of z_exposure_ on z_outcome_ for every 1 year increase in time
